# A Clinical Note

**Published:** 1887-10

**Authors:** George H. Higgins

**Affiliations:** Colorado, Texas


### A CLINICAL NOTE.

Geo. H. Higgins, M. D., Colorado, Texas.

A. B., lawyer, middle-aged, had neuralgia after any prolonged mental effort for ten years. Has tried everything but failed of cure. The pain is in the right side of the temple, in and around the eye, in the ear, and in the lower molar teeth.

*Chewing ice gives some relief.*

℞ Coffea $^{3c}$, one dose, relieved in one hour, and the pains have not returned for the past six months.

